# From Neurons to Cognition: Technologies for Precise Recording of Neural Activity Underlying Behavior

**DOI:** 10.34133/2020/7190517

**Published:** 2020-12-25

**Authors:** Richard H. Roth, Jun B. Ding

**Affiliations:** ^1^Department of Neurosurgery, Stanford University, Stanford, CA 94305, USA; ^2^Department of Neurology and Neurological Sciences, Stanford University, Stanford, CA 94305, USA

## Abstract

Understanding how brain activity encodes information and controls behavior is a long-standing question in neuroscience. This complex problem requires converging efforts from neuroscience and engineering, including technological solutions to perform high-precision and large-scale recordings of neuronal activity *in vivo* as well as unbiased methods to reliably measure and quantify behavior. Thanks to advances in genetics, molecular biology, engineering, and neuroscience, in recent decades, a variety of optical imaging and electrophysiological approaches for recording neuronal activity in awake animals have been developed and widely applied in the field. Moreover, sophisticated computer vision and machine learning algorithms have been developed to analyze animal behavior. In this review, we provide an overview of the current state of technology for neuronal recordings with a focus on optical and electrophysiological methods in rodents. In addition, we discuss areas that future technological development will need to cover in order to further our understanding of the neural activity underlying behavior.

## 1. Introduction

A major question in humanity’s quest to understand the brain is how brain activity encodes information and guides behavior. How do neurons represent our perception of the world around us and then process this information to generate actions that let us interact appropriately with our surroundings? Specific activity patterns of individual neurons and the coordinated activity of populations of neurons can represent sensory information, internal states, behavioral planning, or action execution. Neuronal activity occurs mostly in electric form through changes in membrane potential during action potentials [1]. However, neuromodulatory and intracellular biochemical signaling, which can influence but are not always in sync with the electrical activity of a neuron, can also participate in the encoding of information [2].

In general, measurements of brain activity can be performed across a wide range of temporal and spatial precision using various recording methodologies [3]. The most direct way to measure the electrical activity of neurons is to use electrodes that are implanted into the brain [4]. Using this electrophysiological approach, modern electrodes are capable of recording from hundreds of neurons simultaneously with high temporal resolution. However, electrophysiological approaches have traditionally been limited in their ability to deliver spatial information about the neurons sampled.

In recent decades, optical imaging has emerged as a powerful approach to record neuronal activity in a spatially resolved manner and has been widely used in animal research [5]. Expression of genetically encoded calcium or voltage sensors enables high-precision neuronal activity measurements. Moreover, fluorescent biosensors have been developed to detect other modalities of neuronal signaling beyond voltage and calcium [6]. These novel biosensors can detect the release of chemical neurotransmitters and neuromodulators at synapses as well as measure intracellular signaling pathways, such as kinase activity. These tools will provide a better understanding of how electrical neural activity patterns are formed and altered during behavior. Notably, fluorescence imaging techniques can take advantage of the myriad of genetic tools available to specifically record from a select population of neurons [7].

Electrophysiological and optical recording techniques have been widely used to explore neuronal activity patterns during a variety of behavioral and cognitive tasks. Yet, to understand the relationship between neural activity and behavior, neuroscientists also need methods to precisely measure behavior. The rise of computer vision and machine learning has enabled the development of algorithms to aid with automated measuring and quantification of detailed behavioral actions, such as movement trajectories and kinematics [8]. These new advances will greatly enhance our ability to correlate specific behavioral measures with precise patterns of neural activity.

In the past century, classic *in vivo* recording techniques have led to many landmark discoveries of fundamental neuronal firing properties and helped define how neurons represent information from the outside world [9]. Current technological innovations have further advanced our understanding of how neurons interact in dynamic circuits and how neural activity patterns are involved in behavioral and cognitive processes, such as sensory representation, motor control, or decision-making [10-12]. However, much of how neuronal activity encodes behavior remains unexplored. Though current tools can be employed to tackle many of the open questions, continuous research and development at the intersections of neuroscience, material science, and biomedical engineering will be necessary. New developments will need to expand and optimize the scale, kinetics, and sensitivity of current recording technologies as well as enable the study of other dimensions of neuronal signaling, including neuromodulatory and intracellular signaling as well as the plasticity of neuronal activity.

The need for interdisciplinary collaborations to facilitate such tool development has also been recognized by funding agencies around the world and has been a central focus of several brain initiatives established in recent years [13, 14]. These efforts have already led to numerous technological advances, thus opening the door for future studies of the neural activity underlying behavior.

**Figure 1:**
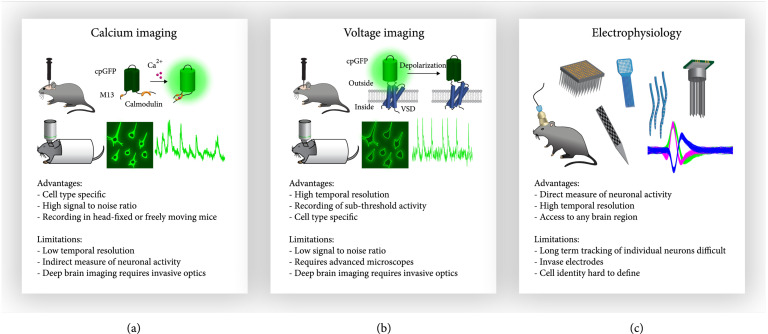
Technologies for recording neuronal activity in rodents. Calcium imaging, voltage imaging, and electrophysiological recordings are three key approaches to measure neuronal activity in rodents. (a) Calcium imaging involves the expression of a fluorescent calcium sensor (GCaMP family shown here), for example, through viral injections in the brain. The fluorescent signal can be monitored through single-photon or two-photon microscopy in freely moving as well as head-fixed animals. Fluorescence intensity traces of neurons reflect their action potential firing. (b) Voltage imaging uses a similar approach by expressing fluorescent genetically encoded voltage indicators instead of calcium sensors. The schematic of ASAP family sensors is shown here. Fluorescence intensity directly indicates neuronal membrane voltage revealing spiking and subthreshold activity. (c) Electrophysiological recordings represent the most direct measure of neuronal activity. Microwire or silicon-based electrodes are implanted into the brain and record voltage changes in freely behaving (shown here) or head-fixed animals. A wide variety of probe designs exist with different geometric shapes and different number of recording channels. Signal traces from electrophysiological recordings need to be computationally associated to individual neurons through spike sorting.

In this review, we present an overview of the current technological developments in the field of optical and electrophysiological neural recordings in awake behaving animals (Figure 1 and Table 1) as well as discuss methods to precisely measure animal behavior. Note that some of these neuronal recording tools can be used for not only passively observing or recording neural activities but also actively intervening neural activities in the brain. While researchers have studied brain activity underlying behavior in a variety of species, ranging from worms to humans, this review will largely focus on tools and studies of rodents. The availability of a powerful genetic toolbox has made the mouse one of the most studied model organisms in neuroscience research. Though more complex model organisms, such as nonhuman primates, possess a more human-like brain structure and display a diverse set of behaviors [15, 16], the design of behavioral studies in rodents is evolving to model more and more complex behaviors and their underlying neural activity [17-21]. In addition, we will discuss the advantages, challenges, and limitations of available methods. We place an emphasis on technologies that are commercially available or accessible through collaborations and, thus, can be implemented in labs not primarily focused on technology development.

**Table 1:** Detailed comparison of various technologies for recording neuronal activity in rodents.

	Ca^2+^ imaging				Voltage imaging		Electrophysiology			
	1P-Ca	1P-Ca-head mounted	2P-Ca	2P-Ca-head mounted	1P-V	2P-V	Microwire electrodes	Silicon electrodes	Next-gen probes	Flexible electrodes
Typical # of neurons/units resolvable	100-1000	100-500^98^	100-500^31,^${}^{*}$	10-100^95^	~10^44^	~10^43^	~20 per probe^115^	~100 per probe^123^	~300 per probe^129^	~15 per probe^136^
Typical temporal resolution	5-25 Hz^†^	5-25 Hz^†^	5-25 Hz^†^	5-25 Hz^†^	0.5-1 kHz^44,45,48^	0.2-4 kHz^43,68^	>1 kHz	>1 kHz	>1 kHz	>1 kHz
Area of tissue damage^‡^	Brain surface	*Φ* 0.5-1 mm hole	Brain surface	*Φ* 1-2 mm hole	Brain surface	Brain surface	*Φ* 0.2 mm hole	<*Φ* 0.1 mm hole	<*Φ* 0.1 mm hole	<*Φ* 0.01 mm hole
Subcellular resolution?	No	No	Yes^30^	Yes^95^	No	Yes	No	No	No	No
Freely moving behavior?	No	Yes^98^	No	Yes^95^	No	No	Yes^115^	Yes^123^	Yes^129^	Yes^136^
Chronic recording?^§^	Yes	Yes	Yes	Yes	Yes	Yes	No	No	No	Yes^134^
Human applicable?	No	No	No	No	No	No	No	Yes^128^	No	No

${}^{*}$While typical 2p microscopes can image field of views with a maximum of 1×1 mm, 2p mesoscopes are capable of imaging large field of views (4×4 mm) resolving up to 3000 neurons simultaneously, though at lower temporal resolution (2 Hz)^71^. ^†^Temporal resolution for Ca^2+^ imaging is limited by slow dynamics of intracellular calcium and calcium indicators. Microscope setups are able to record at higher temporal resolution; however, accurate inference of neuronal spiking is only feasible up to a firing rate of ~25 Hz^31, 106^. ^‡^The area of tissue damage depends on a targeted brain region. Areas stated for Ca^2+^ imaging assumes cortical imaging for 1P-Ca and 2P-Ca and deep brain imaging using GRIN lens implantations for head-mounted imaging. ^§^Capability to long-term track individual identified neurons over multiple days.

## 2. Optical Imaging Approaches to Monitor Neuronal Activity in Awake Animals

Recent decades have seen a rapid development of optical microscopy and molecular sensors that translate neuronal activity into fluorescence signals, propelling the use of imaging approaches for recording neuronal activity in the brain. Early studies used organic fluorescent dyes that report changes in neuronal membrane voltage [22] or changes in the concentration of intracellular calcium as a proxy for neuronal activity [23-25]. Calcium imaging became especially widely adapted with the development of genetically encoded calcium indicators (GECIs) [26, 27]. These allowed for cell type-specific expression and longitudinal recordings of neuronal activity in living organisms. However, calcium is only a secondary readout of neuronal activity and imaging calcium has limitations in terms of temporal dynamics and the ability to detect subthreshold events (Figure 1(a)) [5]. In recent years, the development of genetically encoded voltage indicators (GEVIs) has matured enough to be an alternative, in some cases, to calcium imaging for measuring neuronal activity in a more direct manner (Figure 1(b)). Another recent advancement is the development of fluorescent sensors for synaptic release of neurotransmitters and neuromodulators as well as intracellular signaling molecules, allowing for a more comprehensive understanding of neuronal activity beyond action potentials and calcium signaling. However, one should keep in mind that optical techniques are indirect measures of neuronal activity and are limited by sensor kinetics and efficiency. In this section, we will summarize the most recently developed sensors for imaging neuronal activity in the brain and discuss novel developments in optical microscopy and computational analysis approaches for high-precision measurements of neuronal signals in awake animals.

### 2.1. Genetically Encoded Calcium Indicators (GECIs)

Currently, the most commonly used and well-developed imaging approach to record neuronal activity is calcium imaging. Intracellular calcium signaling plays a central role in a wide variety of cellular processes, and neuronal activity causes a brief influx of calcium, with a single action potential elevating the calcium concentration with a decay time constant of 60 ms. [28] Thus, calcium imaging serves as a good proxy for measurements of neuronal activity, though it should be noted that, due to slow decay constant of cellular calcium, its correlation with electrical neuronal activity is less reliable at higher firing rates. To date, *in vivo* calcium imaging has been used in a variety of studies to unveil the neuronal activity patterns involved in sensory perception, motor control, decision-making, and many other behaviors.

#### 2.1.1. Design and Development of GECIs

**Figure 2:**
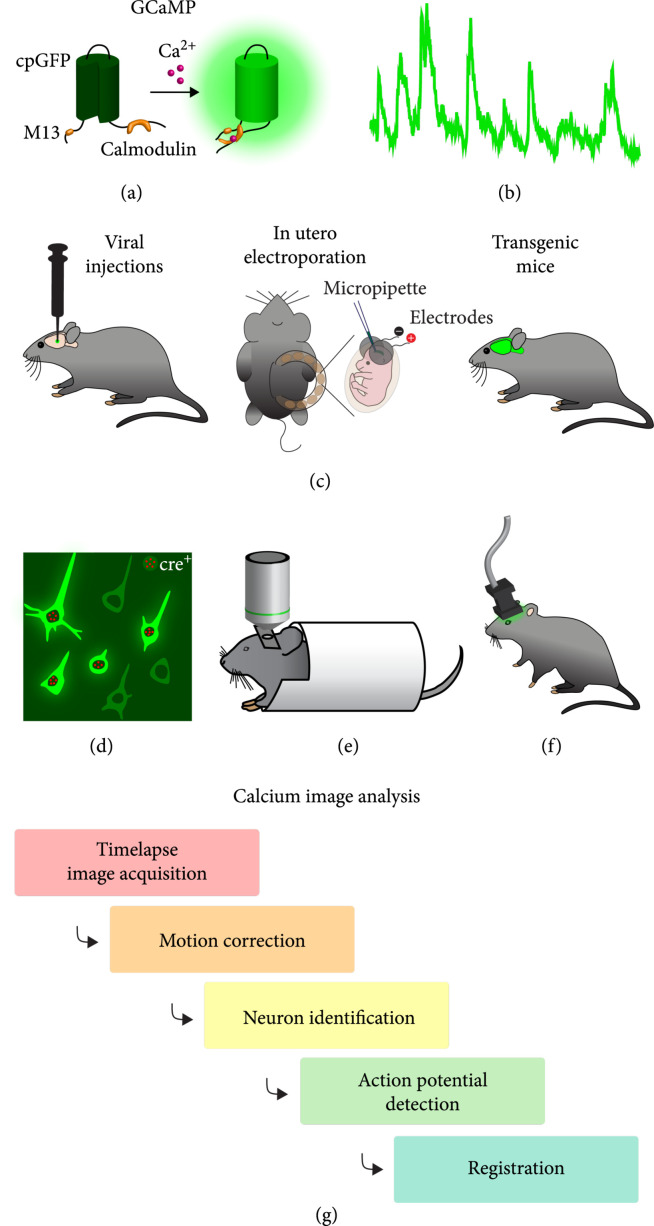
Imaging of genetically encoded calcium indicators. (a) Schematic of GCaMP family of calcium indicators. (b) Example fluorescence trace of a single neuron. (c) Different approaches to express calcium indicators in rodents. (d) Cell-type-specific expression of GECIs in cre-positive neurons. (e) Head-fixed imaging. (f) Imaging in freely-moving mice using head-mounted microscopes. (g) General calcium image analysis pipeline.

The basic design of most GECIs consists of a fluorescence protein fused to the calcium-binding protein calmodulin and a calmodulin-binding peptide (M13). Upon calcium binding, the peptide will bind to calmodulin, causing a conformational change in the fluorescence protein (FP) which enhances its fluorescence signal (Figure 2(a)). In early versions of GECIs, the FP used was a pair of BFP and GFP where the conformational change induced Förster resonance energy transfer (FRET) signaling between the two FPs [26]. In newer GECIs, such as the widely used GCaMP series, the FP is a circularly permuted EGFP (cpGFP) whose fluorescence increases with increased calcium binding [27, 29, 30]. Over the past two decades, these sensors have been continuously evolved through many iterations of mutagenesis, improving their brightness, sensitivity, dynamic range, and kinetics. The most recent iteration of GCaMPs (jGCaMP7 family) can reliably detect single action potentials in cultured neurons with a response amplitude between 20% and 70% ($\Delta F/F_{0}$), and in the visual cortex, jGCaMP7 responds to visual stimulation with 3-10-fold increases in fluorescence *in vivo* (Figure 2(b)) [30]. Another newly developed fast GECI, XCaMP-Gf, can reliably resolve individual action potentials (APs) at firing rates up to 25Hz *in vivo* [31]. Resolving individual APs in neurons with higher firing rates, such as cortical PV interneurons, cerebellar Purkinje neurons, and subthalamic neurons, is more challenging, but XCaMP-Gf is able to detect changes, despite not being able to resolve individual APs, in firing rates up to 60Hz [31].

#### 2.1.2. Cell Type- and Circuit-Specific Expression of GECIs In Vivo

A powerful advantage of GECIs and other genetically encoded biosensors is that their expression can be targeted to a desired brain region or cell population [7]. There are three main ways by which these sensors can be expressed in rodent brains: viral transduction, *in utero* electroporation, and generation of transgenic animals (Figure 2(c)).

The most commonly used method to express GECIs *in vivo* is through stereotactically guided viral injections. Therefore, DNA coding for indicators is packaged in viral vectors, such as adeno-associated viruses (AAVs), and a small amount of virus is pressure injected into brain tissue using stereotactic coordinates to target specific brain areas. By using specific promotors or recombinase systems, specific neuronal populations or circuits can be targeted. Many commonly used constructs are available for purchase from core facilities or private companies. In addition, most of the companies also offer packaging services for labs that are not equipped to prepare custom viruses. The advantage of this approach is that viral injections exhibit the most versatility in targeting the neuronal population of choice.

*In utero* electroporation involves injecting a DNA plasmid encoding the indicator into the brain ventricular system of prenatal or early postnatal animals and using electrical current to cause DNA to enter the target neurons. By taking advantage of the developmental time point and target structure, specific cell populations can be targeted, such as neurons in individual layers of the neocortex, hippocampus, cerebellum, cortical interneurons, or nonneuronal cells such as oligodendrocytes and astrocytes [32]. While this is well suited for studying neuronal circuit activity during development, toxicity caused by prolonged expression of calcium indicators as well as lack of precise control over cell-type specificity and expression levels makes this approach less suitable for studying neuronal activity in adults.

Another popular approach is to use transgenic animals that have been engineered to genomically encode the sensors. Expression is typically driven in a conditional manner, for example, controlled by the cre-loxP recombinase system, such as in the Ai148 mouse line expressing GCaMP6f [33]. Similar to viral delivery, this system allows for selective expression of sensors in selected neuronal populations and provides the additional advantage of homogenous expression levels across individual neurons. However, toxicity arising from prolonged expression can pose a problem [34].

A major advantage of using genetically encoded biosensors is the ability to monitor the activity of a specific neuronal population that is of interest for a given scientific question (Figure 2(d)). Conditional expression of sensors using the cre-loxP system represents a powerful tool to target genetically defined cell populations, and a myriad of cre-driver transgenic mouse lines has been generated in the past years that can be readily obtained. Additionally, newly developed viral serotypes enable targeting of neurons that are part of specific neuronal circuits with defined inputs or outputs. Retrograde viruses, such as rAAV2-retro [35] or CAV2 [36], allow for expression of sensors in projection neurons by injecting virus in the target brain structure. To specifically label neurons that receive input from a defined neuronal population, one can take advantage of the anterograde transsynaptic properties of AAV1 [37]. Injection of AAV1-cre in projection neurons and cre-dependent biosensors in their target structure allows for recording of neuronal activity in neurons with defined inputs.

#### 2.1.3. Multiplexing Neural Recordings with Multicolor GECIs

A powerful benefit of optical imaging is the ability to simultaneously acquire images in multiple colors. While most image acquisition systems can image two colors, typically green and red emission, imaging of up to four colors or more is feasible. The majority of currently available sensors are based on GFP and consequently have green emission spectra [29, 30]. However, red GECIs are also being rapidly developed and possess characteristics that make them well suited for *in vivo* imaging [31, 38, 39]. By combining two independent recombinase systems (cre-loxP and Flp-FRT), these red and green GECIs can be expressed in two distinct populations of neurons, allowing the study of their interactions and contributions to behavior [31]. While not as mature as green or red indicators, blue, cyan, and yellow GECIs have also been developed [31, 40]. This multiplexing approach also provides opportunities to simultaneously record multiple signaling modalities, such as combining calcium imaging with imaging of neuromodulator signaling.

#### 2.1.4. Imaging Compartments of Neurons

Compared to other neural recording approaches, optical imaging provides the spatial resolution to record the activity of separate cellular compartments within neurons. Imaging of axons and axonal boutons, for example, can prove powerful to study the outputs of neurons. In some neuron types, regular GECIs do not traffic well into axonal compartments. However, this issue can be overcome by using a small peptide tag to enhance axonal localization, such as in axon-GCaMP [41]. On the other hand, to study how individual neurons receive different inputs, one can image calcium activity in dendrites and dendritic spines. Most GECIs express well in dendrites [29]; however, sparse expression and low baseline fluorescence make tracing of dendritic segments challenging. Combining green calcium imaging with a red cell morphology marker, such as dsRed or tdTomato, or using a GECI variant with higher baseline fluorescence, such as GCaMP7b [30], can overcome this issue. Conversely, if the goal is to use calcium activity as a proxy for action potentials, signals arising from other background neuronal processes can complicate quantification, especially with one-photon imaging. Recently developed versions of GECIs that are targeted to the soma can be a solution to this problem [42].

### 2.2. Genetically Encoded Voltage Indicators (GEVIs)

In recent years, newly developed genetically encoded voltage indicators have become increasingly useful for *in vivo* imaging of neuronal activity in behaving animals. Recording changes in membrane voltage is the most direct readout of neuronal activity and has a temporal advantage over calcium imaging in that it does not rely on a slow second messenger. It also has the ability to detect subthreshold and hyperpolarizing signaling events. However, the faster kinetics of voltage dynamics compared to calcium dynamics also represents a challenge, requiring faster imaging systems and brighter fluorophores. Currently, a wide variety of protein designs exist for engineering GEVIs, usually consisting of one voltage-sensing domain and a fluorophore domain [5]. The voltage-sensing domain can be a microbial rhodopsin or the voltage sensitive domain of voltage-sensing phosphatases (VSP). In the case of opsin-based GEVIs, the intrinsic fluorescence properties of the opsin can be used as a fluorophore domain, or an additional fluorophore is fused to the opsin, and voltage-dependent FRET signal between this fluorophore and the opsin is read out for imaging. In the case of VSP-based GEVIs, a fluorophore domain is needed, such as the FP cpGFP. Voltage-dependent conformational changes in the voltage-sensing domain then leads to changes in fluorescence of the FP. To increase the signal to noise ratio, GEVIs have been fused with a soma-localization peptide, to enrich its expression in the soma and limit neuropil contamination [43].

“SomArchon” [44] and “QuasAR3” [45] are recent developments of opsin-based GEVIs using the intrinsic fluorescence properties of the opsin. They are excited by red light and emit in the near-infrared spectrum with submillisecond response kinetics; therefore, they are well suited for one-photon imaging with excellent temporal resolution to resolve single APs. A major drawback of this type of GEVIs is their dimness, which requires high excitation powers and may result in bleaching. Moreover, these sensors cannot be used in two-photon imaging due to their absorption properties. To overcome these issues, several GEVIs have been developed that have a second fluorophore fused to the rhodopsin domain. In “Ace2N” [46], for example, the FP mNeonGreen has been fused to a bacterial rhodopsin, allowing for voltage-dependent FRET signal changes. This approach combines the fast kinetics of rhodopsin with the brightness of FPs. However, so far the degree of modulation, especially under two-photon excitation, and photostability can be limiting [47]. A different fluorophore approach was taken for the GEVI “Voltron” that entails a HaloTag dye-capture protein domain fused to the opsin [48]. The HaloTag domain can bind to a bright and photostable organic fluorescent dye (Janelia Fluor) that is exogenously added for imaging. Although not suitable for two-photon imaging, Voltron presents one of the brightest and most photostable GEVIs to date and allows for a large field of view imaging of APs and subthreshold neuronal voltage signals.

The “ASAP” family of voltage sensors is a prominent member of GEVIs consisting of the voltage-sensing domain of VSP fused to a FP, in this case cpGFP. The newest member of this family, “ASAP3” [43], is well suited for 2p imaging *in vivo*. While it has slightly slower kinetics than opsin-based GEVIs, it can resolve single action potentials *in vivo* with fluorescence changes in the order of 5-10%.

### 2.3. Genetically Encoded Neurotransmitter and Neuromodulator Indicators

Calcium and voltage imaging aim to monitor spiking activity of neurons, arguably the most visible form of information transfer in the brain. Yet, communication between neurons is mostly chemically mediated through neurotransmitters, such as glutamate and GABA, and neuromodulators, such as dopamine, acetylcholine, norepinephrine, and serotonin. While fast neurotransmission is mediated by ionotropic neurotransmitter receptors, neuromodulator signaling is typically mediated by G-protein-coupled receptors (GPCRs). Recently, there has been enormous progress in the development of fluorescent indicators for neurotransmission and neuromodulation as well as downstream intracellular signaling pathways. A particularly successful design approach is to fuse cpGFP with proteins binding specific neurotransmitters. As with GECIs and GEVIs, neurotransmitter binding will cause a conformational change that affects cpGFP fluorescence. Using bacterial glutamate- or GABA-binding proteins, the glutamate and GABA sensors “iGluSnFR” [49] and “iGABASnFR” [50] have been created and successfully used for imaging *in vivo*.

A similar approach has been used to develop sensors for dopamine (“GRAB_DA_” [51] and “dLight” [52]), acetylcholine [53, 54], norepinephrine [55], and serotonin [56]. These sensors typically use mammalian GPCRs that bind the specific neuromodulator, for example, D1 or D2 dopamine receptors for dopamine sensors, with cpGFP inserted at an intracellular loop of the GPCR. To measure the downstream intracellular signaling pathways activated by neuromodulators, several groups have recently developed fluorescent kinase sensors that, for example, can detect changes in protein kinase A activity [57, 58] or cyclic AMP response element-binding protein (CREB) [59].

In addition to these genetically encoded indicators, a few other fluorescent neuromodulator sensor approaches have been developed. For example, implanting HEK293 cells that express fluorescent neuromodulation sensors [60] or carbon nanotubes with neuromodulator binding ability [61, 62] represents alternative approaches to monitor neuromodulation signaling activity *in vivo*. For a full review on current optical approaches to measure neuromodulation, see [6].

### 2.4. Advances in Optical Microscopy

Advancing optical microscopy technology for fluorescence imaging has been a major focus of recent developments in neuroscience and biomedical engineering. The rapid expansion of new optical bioindicators has generated a need for advanced microscopy techniques, pushing the boundaries of engineering, optics, and physics. There is a large variety of microscopes and optical approaches currently available for neural imaging *in vivo*, ranging from relatively simple widefield microscopy to multiphoton imaging and complex optical arrangements allowing for high-speed, deep-brain, or multiregional brain imaging. To monitor neuronal activity in behaving animals, either the animal’s head needs to be fixed to the microscope or the microscope needs to be miniaturized to be mounted on the head of a freely moving animal (Figure 2(e) and (f)). For a more detailed review, see [63, 64].

#### 2.4.1. Widefield Microscopy

One of the simplest microscopy setups to monitor brain activity *in vivo* is the widefield epifluorescence microscope. While this can provide single-cell resolution, it is restricted in the imaging depth due to the scattering nature of brain tissue. Today, *in vivo* widefield microscopy is mostly used for low magnification cortex-wide imaging or is used in miniature microscopes that enable imaging of freely moving mice (see respective sections below).

#### 2.4.2. Two-Photon Microscopy through Cranial Windows

The most commonly used optical microscopy technique for monitoring brain activity is two-photon (2p) laser scanning microscopy. Compared to one-photon widefield imaging, 2p imaging allows for deeper tissue penetration, making it well suited for imaging of cortical neurons up to several hundred micrometers deep from the brain surface. The use of longer wavelengths (~920nm for GFP excitation) and absence of out-of-focus excitation enables efficient tissue penetration with reduced scattering.

For imaging in mice, a craniotomy is performed to gain optical access to the brain. This surgical process involves removing a small piece of the skull above the imaging region of interest and replacing it with a small glass cover slip. Craniotomies are typically round or square with a diameter of 2 mm-5 mm. Conventional 2p microscopes can image individual field-of-views of up to 1 mm×1 mm with cellular resolution. To image subcellular structures such as dendritic spines or axonal boutons, further magnification is required, resulting in field-of-views of around 100 μm×100 μm.

Functional imaging of neuronal activity with calcium indicators typically requires acquisition speeds of tens of frames per second, which can be achieved with 2p laser scanning microscopes equipped with resonant scanners. While most studies focus on imaging neurons in single z-planes, some studies require monitoring of neurons in a three-dimensional volume. Traditional microscope objective stages move too slowly for reasonable volume imaging rates. However, using piezoelectric-driven z-stages allows for imaging of a 25 *μ*m deep volume at 7-8 volumes per second [65]. Furthermore, approaches using beam shaping through electrical lenses and mirror arrays or remote focusing [66] allow for three-dimensional volume imaging without physical movement of the objective. Alternatively, volume imaging can be achieved by elongating the excitation laser beam such as by using a Bessel beam focus. This approach essentially images the 2D projection of a 3D volume and is particularly useful in sparsely labeled samples where functional time-lapse images can be reconstructed onto the still image of the 3D structure [67].

To image neuronal activity using voltage indicators, their faster dynamics require higher imaging speeds. By reducing the resolution and field of view, conventional 2p microscopes with resonant scanners can achieve imaging speeds of up to ~200 frames per second [47]. However, specialized microscopy systems now enable 2p imaging of voltage indicators at kilohertz rates [43, 68].

#### 2.4.3. Imaging Multiple Brain Regions

In recent years, it has become more and more apparent that understanding neural activity patterns underlying any given behavior or animal cognition requires the study of how neuronal activity in multiple brain regions interacts with each other. This in turn requires technical approaches to monitor brain activity in multiple brain regions simultaneously. Low magnification wide-field microscopes have been used to measure bulk neuronal activity across the entire cortical surface [69, 70]. However, this approach lacks the resolution to visualize activity of individual neurons.

Typical 2p microscopes with cellular resolution, on the other hand, are limited to imaging single field of views with a maximum of ~1 mm diameter. There have been several approaches to develop novel microscopes that allow for imaging of larger fields of view or multiple smaller fields of view simultaneously. The “Mesoscope” [71] and the “Treapn2p” microscope systems [72] combine a novel scanner and objective optics to enable imaging of FOVs up to 4 mm×4 mm at up to 2 frames per second at cellular resolution. These systems also allow for simultaneous imaging of multiple smaller FOVs within the large objective FOV at frame rates up to 30 frames per second.

While a Mesoscope makes imaging of adjacent cortical regions such as motor and somatosensory cortex or primary visual and higher visual areas possible, the size of the FOV is still limited by the single objective. To measure interactions between brain regions that are further apart from one another, recent designs have developed a dual-arm microscope with two independent optical scan mechanisms [73]. This enabled studying the interactions of cortical and cerebellar neuronal activity during motor behavior in mice [74].

Another approach to record the interactions of local neuronal activity and cortex-wide network dynamics is to combine high-resolution 2p imaging with low-resolution widefield microscopy [75]. This multiscale approach allows for the analysis of how the activity of individual neurons in a brain area correlates with cortex-wide bulk activity dynamics.

#### 2.4.4. Deep Brain Imaging

Due to the optical scattering properties of the brain, most early imaging studies have been focused on the cortex. Even with 2p excitation, it is difficult to penetrate more than 500 *μ*m into brain tissue preventing the study of deep-cortical layer neurons or subcortical brain structures.

A simple way to gain optical access to deep brain structures is to remove the superficial brain tissue through aspiration and insert a cannula with a coverslip at the bottom end. This approach can be used to reach brain regions such as the hippocampus [76, 77] or dorsal striatum [78, 79] but is limited by the size of excavation (typically 2.5 mm diameter) and achievable depth of around 1.5 mm.

Implantation of microoptics in the brain can reduce the impacted area and reach deeper brain regions. For example, by inserting a microprism (1.5 mm side length), one can access regions such as mPFC and MEC which are part of the cortex that lie over 1 mm deep from the surface [80]. Prisms typically reflect light at 90 degrees, such that the target brain region is imaged from the side. This not only allows for imaging of otherwise hard to reach areas but also enables imaging of entire cortical columns in one field of view [81].

Gradient index (GRIN) lenses are long cylindrical lenses that reflect the light from the objective to create a new focal plane on the other end of the GRIN lens [82, 83]. Implanting GRIN lenses into the brain allows for imaging of deeper brain regions. These lenses are typically between 0.5 mm and 1 mm in diameter, up to 10 mm long, and can be directly inserted into the brain or mounted through a guide cannula with a glass cover slip the bottom. While GRIN lens implantation still requires aspiration of tissue or tissue displacement using a guide needle, this approach is less invasive than direct excavation and allows for imaging of brain structures as deep as the amygdala or hypothalamus [84-86].

Apart from implanting optics in the brain, recent efforts have also focused on optimizing the excitation laser beam to penetrate deeper. One such approach is to compensate for aberrations of the laser wavefront caused by inhomogeneous diffraction in brain tissue by integrating a deformable mirror such as a spatial light modulator (SLM) in the microscope light path [87, 88]. This allows for accurate measurements of calcium activity in deep layers of the cortex [39, 89].

Another innovation enabling deeper brain imaging is the use of three-photon (3p) excitation microscopy. By using special lasers capable of generating longer excitation wavelengths of 1300nm for green fluorescent proteins or 1700nm for red fluorescent proteins, 3p microscopy reduces the attenuation of the excitation light by brain tissue and requires the coincident excitation of three photons to emit signal, thereby further limiting the excitation volume around the focal plan and reducing out-of-focus background signal [90]. This technology enabled imaging of calcium activity in the CA1 region of the mouse hippocampus using GCaMP6s through a regular cranial window [91], or cortical neuron activity through the intact skull [92].

#### 2.4.5. Imaging Freely Moving Mice

The majority of imaging technologies mentioned above require large microscope equipment, making it necessary to fix the mouse head under the microscope objective (Figure 2(e)). This can be combined with a variety of head-fixed behaviors where mice are able to move their forelimbs or run on treadmills and respond to sensory stimuli. However, the head-fixed setting cannot capture many intricacies of behaviors in freely moving mice. To enable imaging of freely moving mice, several groups have developed miniature microscopes that can be mounted on the head of mice while only minimally disturbing their natural behavior (Figure 2(f)). These miniature microscopes can be categorized into fiber-coupled microscopes, which have an external light source coupled to the microscope optics mounted on the mouse head with flexible light fibers [93-97] and fully contained miniature fluorescence microscopes that include a light source, detectors, and all optics and are only connected through a data cable [98, 99]. While the latter is limited to single-photon excitation, it is the most commonly used approach for calcium imaging in freely moving mice and has been used to record neuronal activity in a wide variety of behaviors including feeding, spatial learning, and fear memory [100-102]. Fiber-coupled microscopes on the other hand allow for the use of 2p [95] or 3p excitation [97] and can provide higher resolution images, enabling the imaging of neuronal activity in subcellular compartments such as dendritic spines in freely moving animals [95].

### 2.5. Image Analysis

Time-lapse image videos acquired through these imaging approaches require specialized software tools to extract activity signal traces and infer action potential firing patterns from individual neurons. While especially evident in calcium imaging, both calcium and voltage imaging signals are slower than the electrical activity of the neuron; thus, signals from multiple APs can overlap. Moreover, when densely expressed, the fluorescent signal from neighboring neurons can spatially overlap. To overcome these issues and reliably detect neuronal activity or APs from individual neurons, computational tools are necessary. Many such tools have been developed, ranging from manual annotation approaches to automated algorithms. While the development has focused on calcium imaging datasets, most of these approaches can be used for other imaging modalities as well. Generally, image analysis requires several steps: motion correction, neuron identification, action potential detection, and registration across imaging sessions (Figure 2(g)). Motion correction is necessary to correct for small movement artefacts that occur during imaging. The main step is the identification of neurons and association of fluorescent signal with individual neurons. This can be either done manually by defining region of interests (ROIs) or with (semi)automated algorithms [103, 104]. For some studies, analyzing this raw calcium or voltage signal trace is sufficient, while for others extracting action potential spike times of a neuron is needed. Here, a set of algorithms can be used to infer neuron spiking from the fluorescence traces [105]. For calcium imaging with recent sensors, individual APs can be reliably extracted up to a firing rate of ~25 Hz. Due to the slow kinetics of calcium sensors and the slow kinetics of the cellular calcium concentration, accurate inference of neuronal spiking at higher firing rates is challenging [31, 106].

Lastly, if the same set of neurons is recorded across multiple separate imaging sessions, a registration is needed to identify and align individual neurons across sessions [107]. While these represent the general steps involved in calcium imaging analysis pipelines, further reviewed here [108, 109], it is worth noting that as each microscope setup has its unique advantages and limitations, the algorithmic approach used will need to focus on the unique properties of the acquired signals, such as the spatiotemporal dynamics, signal sparsity, and signal to noise ratio.

### 2.6. Limitations of Optical Recording of Neuronal Activity

The combination of a large selection of genetically encoded fluorescent biosensors and advanced microscopy represents a powerful approach to study neuronal signaling activity during behavior, yet there are several limitations. First, regardless of imaging modality (calcium, voltage, or other neuronal signals), a sensor needs to be expressed in neurons to transform and report these signals. This transformation is not loss-free and has limitations in temporal dynamics as well as signal intensity. Moreover, overexpression of these exogenous biosensors can cause alterations in normal cell physiology by interfering with the signaling pathway they are measuring. This issue becomes especially important to consider with prolonged expression, such as in transgenic mice [34]. Second, depending on the imaging method, this approach can be severely invasive and can cause damage to the brain region of interest or surrounding brain areas. While imaging cortical neurons through cranial windows is less invasive than deep brain imaging, which requires excavation of superficial brain tissue, both methods can cause inflammation and glial activation. Thus, a recovery period of several weeks is typically required after surgery. Third, imaging of dim fluorescent signals from suboptimal sensors can require high laser powers, which can cause heating and damage to brain tissue [110]. Conversely, water immersion imaging can also cause transient brain cooling affecting cerebral blood flow [111]. While these limitations might not play a role in all situations, depending on the scientific study, they need to be considered and controlled for.

## 3. Techniques for Large-Scale Electrophysiological Recordings

Arguably, the most direct method to measure brain activity is recording electrical signals using electrodes. For over a century, scientists have used electrophysiological recordings to study how neurons communicate with each other and encode information. Throughout this time, continuous technological advancements have improved recording probes to allow for recording of neuronal activity of hundreds of neurons at high temporal resolution and in a variety of brain areas (Figure 1(c)).

**Figure 3:**
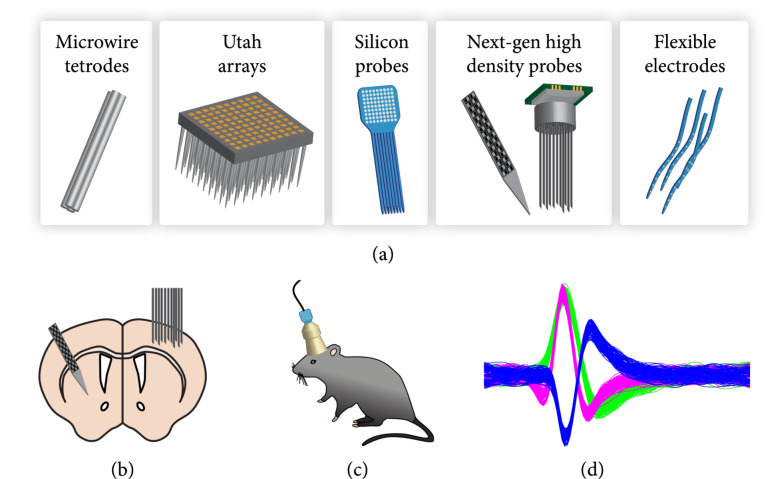
Electrophysiological recordings of neuronal activity. (a) Different types of electrophysiological recording probes. (b) Schematic of next-generation high-density probes inserted into the cortex and subcortical structures of the mouse brain. (c) Schematic of a silicon probe mounted on the mouse head allowing for recording during freely moving behaviors. (d) Signal traces from electrophysiological recordings associated with three individual neurons (green, magenta, and blue) based on their waveform.

Over the years, a wide variety of approaches have been developed to measure electrical activity in the brain (Figure 3(a)). These range from noninvasive scalp electrodes used for electroencephalogram (EEG) recordings to arrays of microelectrodes that are inserted into the brain to measure single neuron activity. Scalp electrodes to measure EEG and brain surface electrodes to measure electrocorticography (ECoG) are used in human patients to measure brain activity in disease states such as epilepsy and have shown promise for use in brain machine interface (BMI) devices. However, these lack spatial resolution and cannot record the activity of individual neurons [112], making them less useful for investigating precise neural activity patterns during behavior.

*In vivo* recording of electrical activity from individual neurons began with microwire electrodes [113], which have been continuously developed over the past decades and are still in use today. With the rise of silicon microfabrication, a new generation of silicon probes was created which allowed for higher density of recording sites. In recent years, this technology has been taken one step further by integrating Complementary metal-oxide-semiconductor (CMOS) technology which allows for active signal processing in the recording probe resulting in even larger numbers and higher densities of recording sites. In this section, we will summarize current technological developments enabling large-scale electrophysiological recordings in rodents during behavior.

### 3.1. Microwire Electrodes

The simplest brain electrode consists of an insulated metal (e.g., tungsten) microwire [113]. These have been used to perform single unit recordings and have led to important discoveries such as receptive fields and orientation tuning in the visual cortex [9]. The introduction of stereotrodes and tetrodes enabled simultaneous recording of up to 20 neurons from a single probe [114-116]. Tetrodes, for example, consist of four insulated wire electrodes twisted together to form four closely placed recording sites. Each of these sites is wider than the sharp tungsten electrodes and records signals from multiple nearby neurons. Electrical signals from individual neurons will differentially contribute to the signal recorded at the four electrodes of the tetrode depending on the spatial location. This allows for post hoc discrimination of these neurons by the waveforms of their signals. Both tungsten electrodes and tetrodes are still widely used for neural recordings in animals. Usually, an array of 20-40 electrodes/tetrodes are inserted into the brain, which can record from several hundred neurons [117-120].

### 3.2. Silicon-Based Electrodes

One major limitation of microwire electrodes is the small number of neurons that can be recorded simultaneously. In order to record a larger number of neurons, one would have to insert larger numbers of electrodes, which can cause severe brain damage at diameters of 30-50 *μ*m per electrode/tetrode. The use of silicon lithography enabled fabrication of more densely packed probes, providing more recording sites at similar probe shank dimensions. These “Michigan-style” probes (first created at the University of Michigan) [121, 122] came in different shapes and sizes but today typically consist of one to eight probe shanks with up to 64 recording sites per shank [123, 124].

A variation of these silicon probes are Utah arrays that consist of a 10-by-10 array of needle-like silicon electrodes [125, 126]. These probes have been especially popular for neural recordings in primates and humans [127, 128].

### 3.3. Next-Generation High-Density Probes

With the increased density of recording sites on neural probes comes a need for increased numbers of connections from the probe, which becomes the next limitation. By integrating CMOS technology, new probes were developed that include electronic circuits used for multiplexing of signals and simplifying its output connections, thus allowing higher density placement of recording sites. One of the most popular of these probes is the “Neuropixels” probe that possesses 960 sites on a 70 *μ*m wide and 10 mm long probe shank. Through multiplexing technology, 384 of these channels can be recorded from simultaneously [129]. The “NeuroSeeker” probe follows a similar technical approach, providing 1356 recording sites that can be recorded from simultaneously [130]. The single shank design of these probes allows for recording of neuronal activity in a large number of neurons along a single one-dimensional axis which could span multiple layers of cortex or deeper brain regions and the cortical area above (Figure 3(b)). By combining multiple such probes, one can sample neuronal activity in a greater variety of brain regions. However, dense recordings along a single plane, such as from neighboring cortical columns, are not possible. To achieve this, a recent study combined mircowire bundles and CMOS sensor chips to create a densely packed array of 15 *μ*m diameter microwires spaced at 100 *μ*m and covering an area with 2 mm diameter (Figure 3(b)) [131]. This approach enables recordings over several hundred sites covering a large area at high density.

### 3.4. Flexible Electrodes

While microwire and silicon-based probes are generally stiff and can cause damage to the brain during movement, another recent development has been the design of flexible electrodes that integrate into the brain tissue. These flexible thread-like probes are typically based on polymers or carbon nanotubes with diameters as low as 1 *μ*m and can have 1 to 64 recording sites per thread [132-137]. Given the flexible nature of these probes, tissue insertion can represent a problem, which can be solved by syringe injection [138], needle punch [132, 135], stiffening of probes through freezing [133], or microfluidics [139]. Yet, these flexible probes are still in the early stages of their development, and further optimizations are necessary for widespread use.

### 3.5. Cell Type Identification during In Vivo Electrophysiological Recordings

One major drawback of electrophysiological recording approaches compared to optical imaging approaches is the lack of ability to select the source of signal. Electrodes will record signals from all surrounding neurons while optical probes can be selectively expressed in specific cell populations. Traditionally, electrophysiologists have been able to distinguish between broad cell types, such as excitatory and inhibitory neurons in the cortex, by comparing their action potential waveforms in recordings. However, this is not a precise measure and cannot distinguish between cell types with similar electrophysiological footprints.

A creative solution to this issue was the combination of optogenetic stimulation with electrical recordings in a method termed “optotagging.” Light-gated cation channels, such as channelrhodopsin-2 (ChR2) [140], can be expressed in genetically defined cell populations and used to activate these neurons during electrical recordings. Thus, light-responding neurons are part of the tagged cell population and can be differentiated from remaining cells during actual recordings [141, 142]. Such approaches have been used to identify PV-positive inhibitory neurons in the cortex [143], direct or indirect pathway striatal output neurons [144], or dopaminergic neurons in the ventral tegmental area (VTA) [145], among others. Typically, neurons are stimulated at the recording site by using either electrodes that combining an optic fiber for photostimulation, so-called “optrodes,” or a separate optic light fiber placed close to the recording electrode [146, 147]. However, this approach can also be used to identify long-range projection neurons by antidromically photostimulating their axons at the target region. For example, pyramidal tract neurons in the motor cortex can be identified by expressing ChR2 in motor cortex neurons and photostimulating their axons in the pons [148].

### 3.6. Isolating Individual Neuronal Signal through Spike Sorting

Most types of modern probes record signal from a large number of neurons with each recording site acquiring signal from multiple surrounding neurons and each neuron contributing to the signal at multiple electrode sites. While such multiunit recordings might provide some information about neuronal population dynamics during behavior [149], understanding the precise neuronal activity patterns involved in behavior involves measuring the activity patterns of individual neurons. To extract single neuron activity information from the recorded data requires software algorithms that identify what component of the signal belongs to which neuron in a process called “spike sorting.” Typically, neuronal spikes are clustered based on parameters such as their waveform to infer their source neuron (Figure 3(d)). Spike sorting software approaches have been developed and optimized over the past decades, and current methods are largely automated [150-152]. For a more detailed review, see [153].

## 4. Precise Analysis of Animal Behavior

One of the biggest questions in neuroscience is how the brain controls behavior. To precisely understand the relationship between neuronal activity patterns and specific behaviors, we need not only tools to record brain activity but also tools to accurately measure and quantify behavioral actions. Nonhuman primates are powerful model organisms to study neural correlates of anthropomorphic behaviors since they are capable of performing complex behavioral tasks, including communication [154], decision-making [155], and complex motor movements [156]. This allows for detailed measurements of diverse behavioral variables that can be correlated with electrophysiological recordings of brain activity. Traditionally, behavioral measurements in rodents have been more limited, and extrapolating from rodent behavior to human behavior needs to be done with care. Yet, in recent years, neuroscientists have developed novel tools, such as computer vision aided tracking, to achieve more precise measurements of rodent behavior [21] enabling a more detailed understanding and characterization necessary for relating behavior to neural activity.

**Figure 4:**
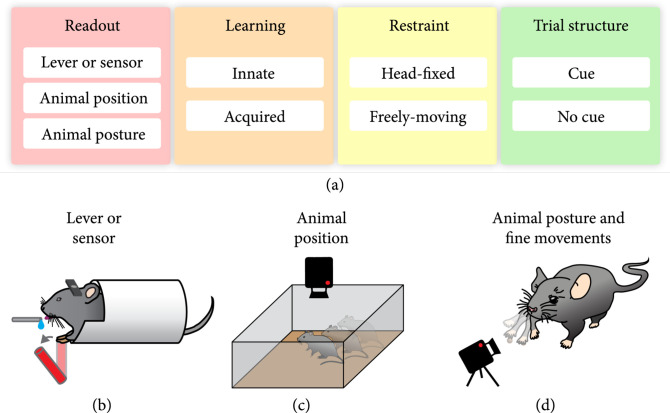
Analysis of animal behavior. (a) Classification of typical mouse behavior tasks based on methods of readout, task learning, animal restraint, and trial structure. (b) Schematic of mouse performing a head-fixed lever-push task for water reward. (c) Schematic of video recording animal positions in an arena. (d) Schematic of tracking animal posture and limb movements in a reach to grasp task.

For simultaneous neuronal activity recording in rodents, behavior tasks can be classified based on four physical and psychological criteria that govern the ease of implementation, types of analysis that can be performed, and which aspects of behavior can be correlated with neuronal activity (Figure 4(a)). First, an important factor to associate behavior with its underlying brain activity is the measurement and quantification of behavior. There are three broad categories of methods that are commonly used to achieve this: recording a lever press or a similar sensor, recording animal position in an arena, and recording animal posture (Figure 4(b)-(d)). Second, behavioral tasks can be further classified by their type of acquisition, i.e., whether a studied task represents a natural and innate behavior, such as eating, or a learned behavior, such as skilled reaching. Third, an important aspect of the behavior for simultaneous recording of neuronal activity is how the animal is restrained. As discussed above, many recording systems, especially microscopes, are large and require the head of the mouse to be fixed in place, while other recording approaches allow for small head-mounted devices to be fixed to freely moving mice. Even with head-fixation, mice are able to perform a wide variety of tasks, though some behaviors can only be observed in freely moving animals. Lastly, behaviors can be classified by their trial structure, for example, whether animals perform a self-initiated task or are guided by a sensory cue. In this section, we will discuss the different methods to measure and quantify behavior and approaches to correlate behavior with neural activity.

### 4.1. Sensor-Based Behavior Readout

A widely used approach to measure animal behavior is to train them to interact with a mechanical sensor whose readout is used as a quantifiable measure of the behavior. These include mice licking for a water reward, pressing a lever or touchscreen for reward, or running on a treadmill [157, 158]. This approach can be used to study the neuronal activity underlying sensory processing and discrimination, decision-making, or motor actions. In their simplest form, these readouts are binary (press/no-press, lick/no-lick, run/no-run), but the sensor mechanisms can be taken a step further and, for example, can be used to measure lever push trajectories [12, 159]. Many sensor-based behaviors have a trial structure consisting of one or more sensory cues, an action phase, and a reward. This allows for measures of neuronal activity in these distinct phases, and through repetition of many trials, trial averages, and intertrial variation can be studied. However, averaging across trials also runs the risk of losing temporal resolution when correlating behavior with neuronal activity, since the timing and temporal structure of task performance can differ between trials and across animals. Either controlling for such variability in the task design or post hoc processing of individual trial data can help overcome such issues [160].

### 4.2. Animal Position Detection

Monitoring animals through videography has been another frequently used method to measure animal behavior. In many behavioral tasks, this has been used to record where an animal is located within a test chamber or arena. For example, studies of the neuronal activity patterns underlying spatial navigation use this method to localize animals while simultaneously recording brain activity [117]. Animal performance in solving different types of mazes has been extensively studied in the context of learning and memory, and open-field exploration or similar behaviors are used to understand anxiety. Moreover, videography is used to measure freezing as a fear response in fear-conditioning assays. These behaviors can only be observed in freely moving mice and are thus most suited for miniature microscope or electrophysiological recordings. However, in recent years, the use of virtual reality has become popular, where head-fixed mice are moving on a treadmill or floating sphere and controlling a virtual environment displayed on computer screens in front of the mice [77]. This has allowed to use the treadmill or sphere movement as a measure for the animal’s position and enabled implementing spatial maze tasks in head-fixed mice.

### 4.3. Animal Posture and Fine Movement Recording

In recent years, the videography of animal behavior has been taken a step further to study precise movement kinematics and dynamics of animals during a variety of behaviors. Traditionally, this has been achieved by using markers placed on animal limbs. However, recent advances in computer vision and deep learning algorithms have facilitated this process and enabled automated, marker-free animal posture detection. Several published software toolkits, such as “DeepLabCut,” are currently available to achieve this and typically require manual annotation of a small subset of the video data to train the algorithm [161-163]. Therefore, users need to mark the body parts of interest in a couple of hundred frames of the video for subsequent automated detection of these body parts in large datasets. Such computer vision approaches have been used to detect rodent paw trajectories and digit positioning during reaching tasks, mouse stride and limb movements during treadmill running, or tongue movements during licking [164-166]. Many of these movements occur at fast speeds requiring high-speed cameras (at least 100 frames per second) to accurately capture the dynamics of these movements. A single camera suffices for 2D posture detection; however, for 3D reconstruction, a depth camera or multiple cameras are needed. Alternatively, a single camera can achieve multiple recording angles through the use of mirrors. Limb position information obtained through automated detection can, for example, construct limb movement trajectories which can be correlated with concurrent neuronal activity. While the behaviors described above are mainly trained behaviors, computer vision approaches have also been used to detect and categorize naturalistic or innate behaviors [17, 18, 167-169].

## 5. Conclusions and Future Perspectives

The development of technology for recording of neuronal activity during behavior has been crucial to neuroscience research and has enabled a wide range of studies that significantly advanced our understanding of the brain. Recent years have seen a rapid rise in new technologies that allow for ever more detailed and comprehensive studies of brain signaling. Optical calcium imaging has served as a powerful approach to record neuronal activity with cell-type specificity, and voltage imaging is gaining traction to become a direct measure of neuronal signaling with high temporal resolution. Electrophysiology has been the core method of recording neuronal activity for past decades and has undergone significant technological improvements, enabling high precision recordings of hundreds to thousands of neurons simultaneously. These methods are opening doors for groundbreaking studies of brain activity and behavior, yet we are only beginning to comprehend how the brain encodes and processes information to achieve behavior and cognition.

The coming years and decades will see a continued growth in new and improved neural recording technologies. In the most obvious direction, we will see further optimization of current imaging and electrophysiological recording techniques in regard to the scale of sampling as well as the kinetics and sensitivity of these recordings. But even with the ultimate goal to record the spiking activity of every single neuron in the brain, we are left with two questions: is it enough to know the activity pattern of all neurons to decode behavioral representation in the brain? And is it even necessary to achieve this goal in order understand behavioral representation in the brain?

Even with the existing sparse recording techniques, neuroscientists are generating more information than we can make sense of. While knowing the activity patterns from more neurons can help our overall understanding, it might only increase the amount of information we cannot comprehend. Ultimately, decoding the brain is as much a challenge in computational and data science as it is neurobiology. Theoretical models will be an important factor guiding us in the quest to decode neuronal activity.

From current experiments, we have already learned that the brain is organized with high redundancy and possesses a large capacity for adaptation. Thus, it might be more important to understand general principles of how neuronal activity is generated and modulated during behavior, rather than achieving comprehensive recording from all neurons concurrently. Notably, to understand these general principles, we will need a better understanding of neuronal activity properties beyond action potentials. Cellular and biochemical signaling plays an essential role in shaping neuronal firing properties, and understanding these signals will be critical to guide the formulation of theories about neuronal activity patterns during behavior and cognition.

To test such theories, we will need to combine neural recording technology with methods to define neuronal populations and manipulate neuronal activity. These approaches are especially important since even optimal large-scale recording of neuronal activity only provides correlative evidence, whereas specific neuronal perturbations are necessary to gain a causal understanding of the role of neuronal activity.

Another important element in using new technology to advance our understanding of the brain will be to ensure accessibility and reproducibility of newly developed tools. With new manuscripts describing technological advances being published every day, we as a community need to define a set of metrics by which these tools can be compared and evaluated. Moreover, a key focus of new tool development needs to be the ease of dissemination to the neuroscience research community and to reduce hurdles of implementation in individual labs. Similarly, data acquired in experiments need to be generated and analyzed in a way that allows for easy reproducibility and comparison [170].

Here, we briefly discuss a few areas that will be the focus of future developments in the coming years.

### 5.1. Simultaneous Brain-Wide Recordings

The majority of currently available technology is aimed at studying neuronal activity in individual brain regions. However, accumulating evidence indicates that many behaviors involve coordinated activity across many brain regions [69, 70]. While functional magnetic resonance imaging (fMRI) is providing brain-wide recording abilities, it lacks the spatial and temporal resolution of modern electrophysiological and optical methods. Recent studies have begun to develop and implement microscopy and electrical recording systems to simultaneously examine activity from many brain regions, but this area still remains largely unexplored. Future studies will need to further improve the technology to simultaneously record from distant brain regions in order to understand the distribution and interaction of brain-wide activity patterns during behavior.

### 5.2. Recording Neuronal Activity across Multiple Scales and Modalities

One approach that can aid with gaining a more comprehensive view of neuronal signaling is to develop methods that enable recording neuronal activity across multiple scales and modalities. This would combine the strengths of individual methods, such as the brain-wide recording ability of fMRI with the cellular resolution and kinetics of electrical recordings. On a technical level, these combinatory approaches can also lead to a better understanding, for example, of the signal sources in fMRI measurements [171].

Additionally, brain function is not only comprised of the electrical activity of neurons but also involves biochemical signaling events such as neurotransmitter and neuromodulator release as well as intracellular signaling. Recent years have seen a rapid development of new biosensors that can detect these biochemical signaling events [6]. However, how these relate with electrical signaling in neurons and with behavior remains unexplored. Future development will need to focus on tools allowing simultaneous recording of neuronal electrical activity with these biochemical signaling events. Given the relative ease of multiplexing multiple recording modalities and access to genetic tools, optical *in vivo* imaging represents the greatest opportunity for such studies. Most current reliable fluorescent sensors are emitting in the green light spectrum, and other color variants will need to be developed and optimized. As an alternative approach, some probes for electrical recordings might also allow for simultaneous *in vivo* imaging and electrical recordings [134].

### 5.3. Defining and Manipulating Cell Populations

The development of tools for recoding neuronal signaling has been accompanied by the development of tools to label genetically defined cell populations and techniques for manipulating their activity. Genetic tools not only enable access to specific cell types [7, 172] but also provide labeling of neurons that are active during particular behaviors [173, 174]. Optogenetic and chemogenetic approaches can be used to activate or silence specific neuronal populations [175, 176] and can play an important role in understanding the causality between neural activity and behavior. Future technological developments will further leverage the power of combining cell-type-specific labeling of neurons with neuronal recording. New studies will also show how transcriptional labeling of active neurons relates to calcium activity of these neurons during behavior. Another powerful approach that will see further development is combining calcium imaging with single neuron optogenetics using SLMs [177-180]. These approaches allow for simultaneous recording and manipulating the activity of individual neurons that are part of an ensemble and measure its effect on population neuronal activity and behavior.

### 5.4. Expanding Neural Recording Technologies to a Wider Variety of Model Organisms

In this review, we have focused on technologies for recording neuronal activity in rodent animal models. The powerful genetic tools available in mice make them a useful model for genetic human diseases and allow interrogation of cell-type- and circuit-specific activity underlying behavior. On the other hand, the murine model has limitations when it comes to simultaneous recordings of larger parts of the brain or reliable modeling of higher-level anthropomorphic behaviors. Neural recording techniques, such as calcium imaging, have been popular in the smaller model organisms *C. elegans*, zebrafish, and Drosophila [30, 181, 182], where whole-brain imaging with cellular resolution is possible [183-185]. Electrophysiological and optical recording is also used in other model organisms, such as ferrets, tree shrews, songbirds, and bats, to take advantage of their specialized brains and study higher order visual functions, communication, and navigation [186-190]. Arguably, the model organism best suited to study brain function underlying human-like behavior are nonhuman primates. While such studies need to take more stringent ethical considerations into account, neural recordings in these animals can provide an understanding of neural activity during behavior most resembling human brain function [191]. Electrophysiological recordings in primates have already led to significant finding regarding higher brain functions, such as communication [154], decision-making [155], and complex motor movements [156, 192]. The recent development of new genetic tools, such as the CRISPR-Cas9 system [193], will expand the genetic toolbox for nonhuman primates in the future. Current efforts have already led to the generation of transgenic marmosets and macaques and even a primate model of autism spectrum disorders [194-196]. Bringing optical recording techniques to nonhuman primates will further facilitate the use of primates in understanding neuronal activity during behavior [197]. Overall, future technological developments will need to expand and optimize neural recording techniques for a wider variety of animal models to leverage the unique strengths of each model.

### 5.5. Plasticity of Neural Activity Patterns

A large focus of current studies has been on studying the spatiotemporal neuronal activity patterns while the animal is performing specific aspects of behavioral and cognitive tasks. While this is providing valuable insight into behavior related neural representation, proper comprehension of brain function also requires an understanding of how neural activity patterns change and adapt with experience and learning. Experience-dependent neuronal plasticity is a hallmark of brain function and plays essential roles not only during development, learning, and memory but also during disease and disease recovery. Thus, future studies and technological advancements should support longitudinal studies of neuronal activity during development, behavioral task acquisition, and disease states. Moreover, to understand the guiding principles of how neural activity patterns are formed, we will need to advance technologies to study the cellular and molecular mechanisms underlying neuronal plasticity in live animals. Recent advances in *in vivo* imaging of intracellular signaling [57-59], neuronal structure [198], and synaptic proteins [199, 200] as well as superresolution imaging in vivo [201] have demonstrated the potential for this field and will need to be combined with neuronal activity recordings in the future.

An often-neglected aspect of studying neuronal activity is the role of glial cells in shaping neuronal responses. Recent studies have accumulated evidence that astrocytes [202], oligodendrocytes [203], and microglia [204] not only play a supportive role but actively participate in modulating neuronal signaling and, thus, may participate in encoding behavioral and cognitive information. Future studies will need to incorporate nonneuronal cells as part of the variables and develop technologies to further study glial signaling.

A deeper understanding of the mechanistic principles underlying neural activity patterns and their ability to change is also crucial for future developments of brain machine interface (BMI) devices. For example, this could allow BMI devices to be designed and implemented in a way that interacts with and capitalizes on the brain’s natural ability to learn.

Overall, further advancing the development of new tools and techniques to record neuronal activity across multiple modalities of neuronal signaling and across large brain areas will be necessary for a more comprehensive understanding of how our brain encodes information about the outside world and governs our behavior. These developments will thrive through innovative collaborations between the fields of biomedical engineering, material science, and neuroscience.
